# Ongoing replication forks delay the nuclear envelope breakdown upon mitotic entry

**DOI:** 10.1074/jbc.RA120.015142

**Published:** 2020-11-23

**Authors:** Yoshitami Hashimoto, Hirofumi Tanaka

**Affiliations:** School of Life Sciences, Tokyo University of Pharmacy and Life Sciences, Hachioji, Tokyo, Japan

**Keywords:** replisome, replication forks, *Xenopus* egg extract, nuclear envelope breakdown (NEB), CDK, Wee1/Myt1, Ara-CTP, Ara-cytidine-5′-triphosphate, CDKs, cyclin-dependent kinases, ex-dCTP, excessive amounts of dCTP, M-CDK, M-phase CDK, M-extract, M-phase extract, MiDAS, mitotic DNA synthesis, NE, nuclear envelope, NEB, nuclear envelope breakdown, p97i, p97 inhibitor, PD, PD166285, Pre-RCs, prereplicative complex, S-extract, S-phase extract

## Abstract

DNA replication is a major contributor to genomic instability, and protection against DNA replication perturbation is essential for normal cell division. Certain types of replication stress agents, such as aphidicolin and hydroxyurea, have been shown to cause reversible replication fork stalling, wherein replisome complexes are stably maintained with competence to restart in the S phase of the cell cycle. If these stalled forks persist into the M phase without a replication restart, replisomes are disassembled in a p97-dependent pathway and under-replicated DNA is subjected to mitotic DNA repair synthesis. Here, using *Xenopus* egg extracts, we investigated the consequences that arise when stalled forks are released simultaneously with the induction of mitosis. Ara-cytidine-5′-triphosphate–induced stalled forks were able to restart with the addition of excess dCTP during early mitosis before the nuclear envelope breakdown (NEB). However, stalled forks could no longer restart efficiently after the NEB. Although replisome complexes were finally disassembled in a p97-dependent manner during mitotic progression whether or not fork stalling was relieved, the timing of the NEB was delayed with the ongoing forks, rather than the stalled forks, and the delay was dependent on Wee1/Myt1 kinase activities. Thus, ongoing DNA replication was found to be directly linked to the regulation of Wee1/Myt1 kinases to modulate cyclin-dependent kinase activities because of which DNA replication and mitosis occur in a mutually exclusive and sequential manner.

DNA replication and mitosis are temporally separated by tightly regulated mechanisms during the cell cycle to ensure that DNA replication is completed before mitosis in eukaryotic cells. There are several types of cyclin-dependent kinases (CDKs) whose activities are upregulated and downregulated during the cell cycle, playing crucial roles in the ordered progression of each cell-cycle event (1). The S-phase CDK, mainly undertaken by cyclin E-Cdk2 in vertebrates, is activated at the onset of the S phase and is active throughout the S phase, triggering the initiation of DNA replication (2, 3, 4, 5). On the other hand, the M-phase CDK (M-CDK), mainly cyclin A/B-Cdk1, is activated at the G2/M transition, promoting mitotic entry of the cell (6, 7). Cell-cycle checkpoints are additional regulatory mechanisms that ensure the start of one cell-cycle event only on completion of the previous event, monitoring various internal and external cellular conditions such as nutrient availability in the G1 phase, damaged DNA throughout the cell cycle, replication progression in the S phase, and spindle assembly in the M phase (8, 9). When replication stress inhibits replication progression and causes stalled replication forks in the S phase, DNA replication checkpoint is activated to stabilize those forks for restart and to suppress CDK activities, new replication initiation, and progression into the G2/M phase (10, 11). The replication checkpoint involves the ATR-Chk1 signaling pathway, where apical ATR kinase activates Chk1 kinase and activated Chk1 inhibits Cdc25 phosphatase through phosphorylation (12). Chk1-dependent inhibition of Cdc25 leads to downregulation of Cdk1/2 activities, as Cdc25 usually activates Cdk1/2 during the S to G2/M phases by removing inhibitory phosphorylation on Thr14 and Tyr15, which are carried out by Wee1 and Myt1 kinases (13, 14). Thus, the ability of CDKs to drive various cell cycle events is dependent on intricate balancing between phosphorylated and dephosphorylated sites.

DNA replication is conducted by the replisome, a large protein complex that contains DNA helicases, DNA polymerases, and many other accessory factors (15). The replisome is built up around the prereplicative complex (pre-RC). The pre-RC is assembled on each replication origin during the late M to G1 phase through a sequential binding of ORC, Cdc6, Cdt1, and MCM2-7 (16). Upon progression into the S phase, the S-phase CDK, together with Dbf4-dependent kinase, phosphorylates and activates several initiation factors, facilitating the formation of bidirectional replication forks and replisomes, where the Cdc45-MCMs-GINS complex and DNA polymerases α/δ/ε act as a replicative helicase and replicative polymerases, respectively (17, 18). Replisomes disassemble during replication termination, fork collapse, and mitotic progression. When two forks converge into a replication termination site in the S phase, catenated sister chromatids are resolved by topoisomerase II and replisomes are disassembled by p97 resolvase that recognizes polyubiquitylated MCM7 (19, 20). In case of defective disassembly in the S phase or stalled forks persisting into the G2/M phases, another backup pathway is activated for replisome disassembly in the M phase that also involves MCM7 polyubiquitylation and p97 activity as those during the S phase (21, 22, 23, 24, 25). The difference between the S-phase and M-phase pathways is that the cullin RING E3 ligase (CRL2^Lrr1^ in metazoa and SCF^Dia2^ in budding yeast) is responsible for polyubiquitylation of MCM7 in the S phase, whereas the RING E3 ligase TRAIP is responsible in the M phase (26). Replisome disassembly during fork collapse is not currently well understood.

DNA replication in principle must be completed within the S phase; however, it was observed that mitotic DNA synthesis (MiDAS) occurred on specific regions of chromosomes such as common fragile sites, especially when exposed to replication stress (27). MiDAS prevents under-replicated DNA from being transmitted into daughter cells, and its absence increases risks for genomic instability such as DNA double-strand breaks (DSBs), chromosome bridges, chromosome mis-segregation, and nondisjunction (28). MiDAS is a kind of break-induced replication carried out by a noncanonical conservative mode of DNA replication depending on Rad52, Mus81, and PolD3 (27, 29). It was recently reported that mitotic replisome disassembly helps promote MiDAS at incompletely replicated regions (30).

Mitotic entry and progression are promoted by M-CDKs with the support of other mitotic kinases such as PLK1 and Aurora-A and Aurora-B (6, 7). The activation process of M-CDK creates an irreversible and bistable switch for mitotic entry, phosphorylating hundreds of substrate proteins that underlie various mitotic events (6, 7). Mitosis is characterized by drastic morphological changes within the cell, such as chromosome condensation, nuclear envelope breakdown (NEB), and spindle assembly. Chromosome condensation is mediated by the coordinated action of condensin I/II complexes and topoisomerase II (31). The NEB starts with disassembly of nuclear pore complexes through CDK- and PLK1-dependent phosphorylation of nucleoporins, followed by depolymerization of the nuclear lamina, release of the nuclear envelope (NE) membrane from chromatin, and retraction of the NE to the endoplasmic reticulum (32).

We previously found that mitotic entry drives replisome disassembly at stalled forks depending on M-CDK activity and polyubiquitylation (23). Other groups also discovered the same phenomenon and identified the responsible enzyme as E3 ubiquitin ligase TRAIP (24, 25). In the present study, we examined what happens when stalled forks are released simultaneously with mitotic entry using a *Xenopus* cell-free system. We found that stalled forks can restart during early mitosis before the NEB, but not after it, partly because of mitotic replisome disassembly; we also found that the released ongoing forks delay the timing of the NEB in a Wee1/Mty1-dependent manner. This mechanism may be intrinsically related to the ordered coupling between DNA replication and mitosis.

## Results

### Stalled replication forks can restart during early mitosis when replication stress is relieved

**Figure 1:**
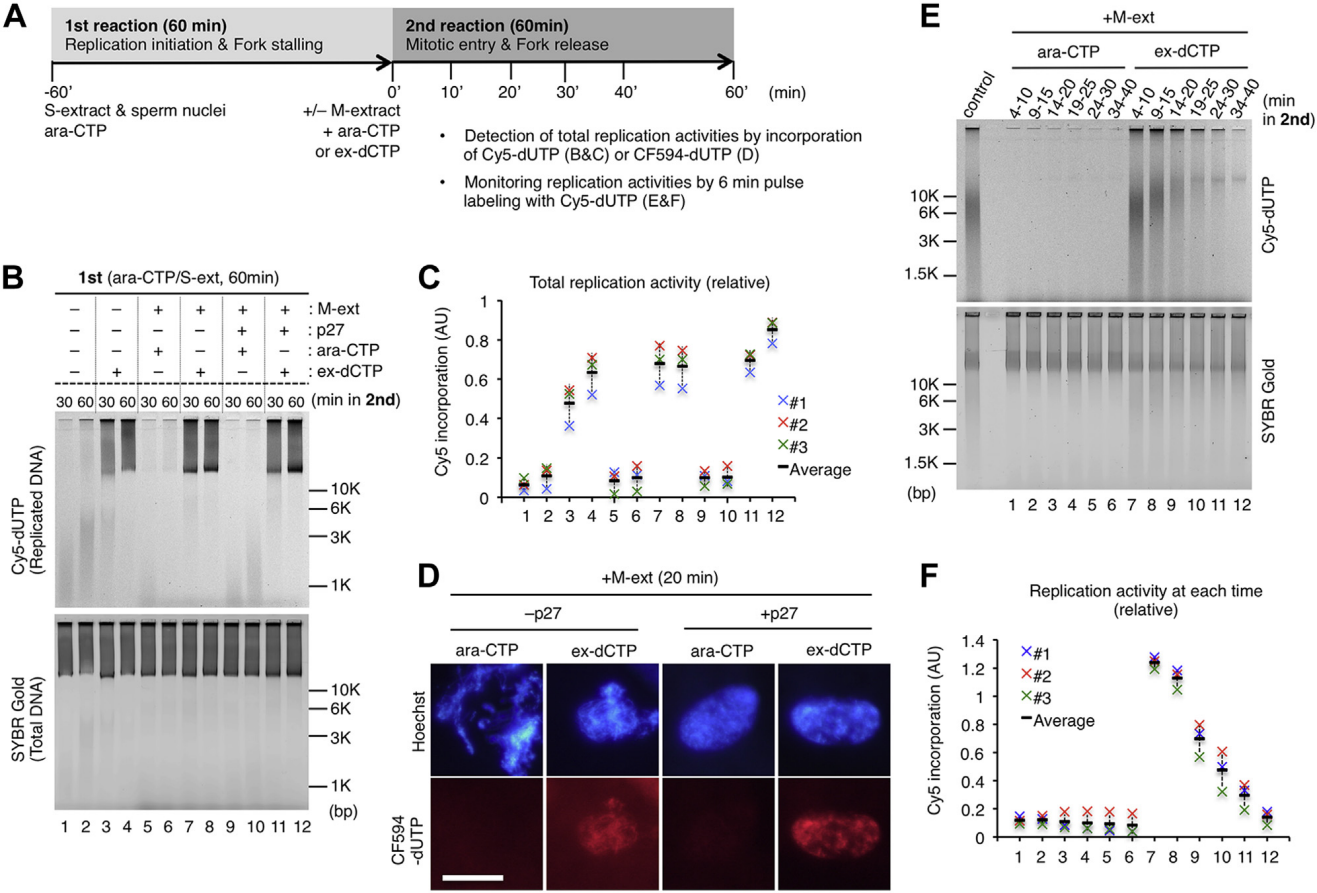
**Stalled replication forks can restart during early mitosis when replication stress is relieved**. *A*, experimental strategy. *B*, in the first reaction, sperm nuclei were incubated in an S-phase extract (S-extract) with Ara-CTP for 60 min. In the second reaction, an equal volume of the M-phase extract (M-extract) containing Cy5-dUTP and Ara-CTP or excess dCTP (ex-dCTP) was added to the first reaction mixture and further incubated for 30 to 60 min. Then, genomic DNA was isolated and subjected to 0.8% TAE agarose gel electrophoresis, followed by SYBR Gold staining. Detected fluorescent signals of Cy5 and SYBR Gold show replicated DNA and total DNA, respectively. Recombinant His-p27 (p27) was added to inhibit CDK activities and mitotic entry. *C*, the same experiment in panel *B* was repeated three times, and the signal intensities of Cy5 were quantified by ImageJ. The replication activity obtained after 90 min incubation in the S-phase extract under unperturbed condition was used as a control for normalization. The relative replication activities were plotted in the graph. Error bar, ± SD. *D*, the same experiment in panel *B* was performed using CF594-dUTP instead of Cy5-dUTP to detect replication activity. After 30 min in the second reaction, nuclei were fixed and observed by fluorescence microscopy. Nuclear DNA was stained with Hoechst 33258. Bar, 20 μm. *E*–*F*, replication activities at each time point were monitored by pulse labeling with Cy5-dUTP added 6 min before isolating genomic DNA, which was analyzed in a similar way as shown in panels *B*–*C*. The replication product at 10 min in the presence of ex-dCTP and p27 was used as the control. Ara-CTP, Ara-cytidine-5′-triphosphate; CDK, cyclin-dependent kinase.

We reported that forced entry into mitosis drives replisome disassembly at stalled replication forks, using *Xenopus* egg extracts with S- and M-phase activities (23). In this study, we first examined whether stalled forks can restart when replication stress is relieved simultaneously with mitotic entry. To this end, sperm nuclei were incubated in an S-phase extract (S-extract) with Ara-CTP to induce stalled forks, to which an M-phase extract (M-extract) was added together with Ara-CTP or excessive amounts of dCTP (ex-dCTP). Their replication activities were monitored through incorporation of fluorescently labeled dUTP (Cy5-dUTP and CF594-dUTP) (Fig. 1). It is known that the addition of ex-dCTP can release forks stalled by Ara-CTP in the S phase (33), and this was confirmed by the results: much stronger replication activities were observed in the presence of ex-dCTP than in its absence, in cases without M-extract or with M-extract whose mitotic CDK activity was inactivated by p27 (Fig. 1*B*, lane 1–4, lane 9–12; Fig. 1, *C*–*D*). We found that ex-dCTP could resume DNA replication even in the presence of an active M-extract as efficiently as in the S phase (Fig. 1*B*, lane 5–8; Fig. 1, *C*–*D*). Fluorescence microscopy observations showed early mitotic nuclear morphology such as condensed chromatin in the absence of p27 (Fig. 1*D*). We also examined the replication activity at each time point by pulse labeling of replication products with Cy5-dUTP (Fig. 1, *E*–*F*) and detected little Cy5 incorporation in the absence of ex-dCTP. In contrast, we found that in the presence of ex-dCTP, higher activities were obtained at earlier time points. These results suggest that stalled replication forks maintain the capability to restart during the early stage of mitotic progression.

**Figure 2:**
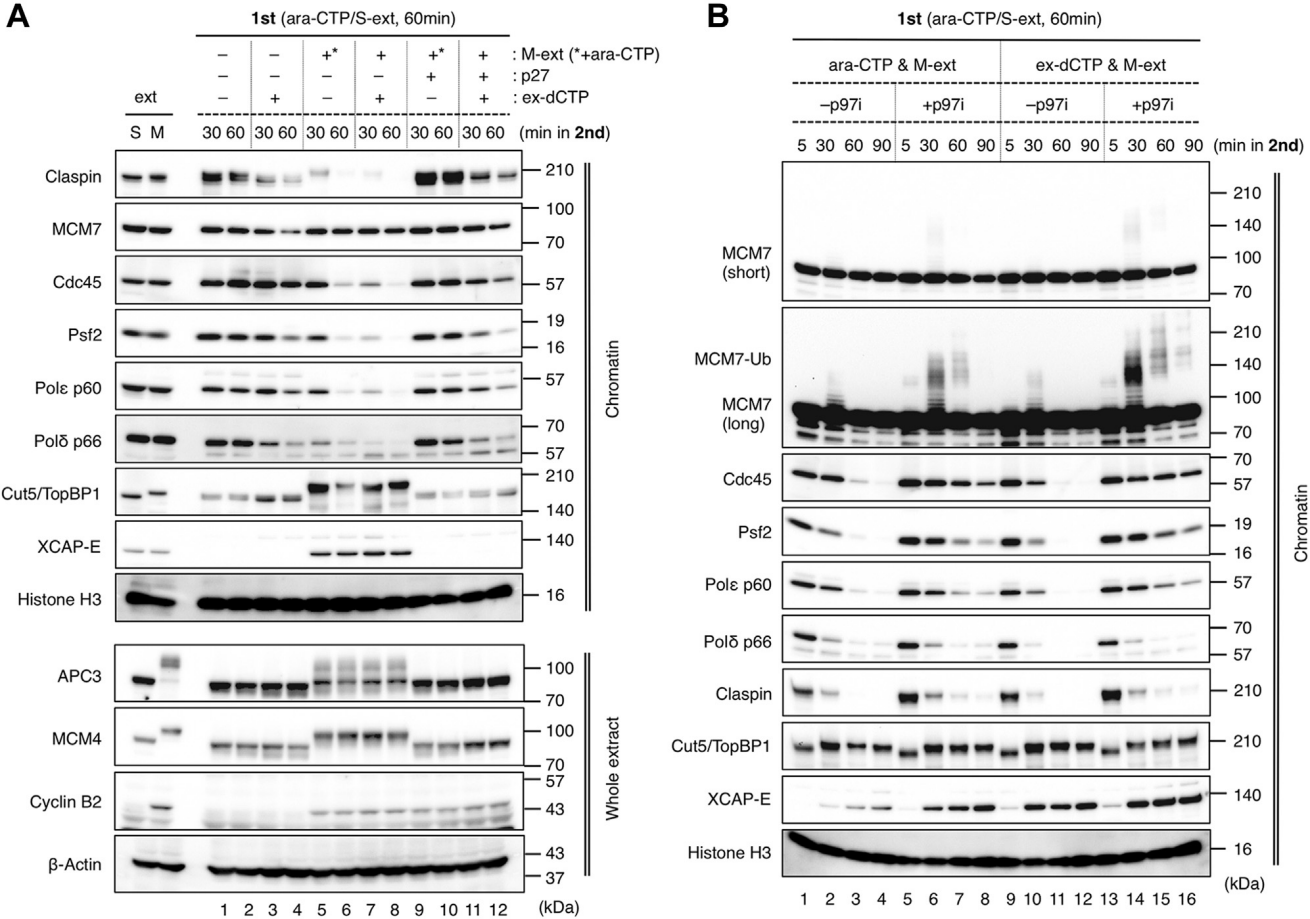
**Mitotic replisome disassembly is mediated by p97 both at perturbed and unperturbed replication forks**. *A*–*B*, the same experiment with Figure 1*B* was performed, and the chromatin fractions and the whole extract were subjected to immunoblotting. In the second reaction as shown in panel *B*, the samples were incubated for up to 90 min in the absence or presence of p97 inhibitor NMS-873 (−p97i, +p97i). Histone H3 and β-actin served as the loading control for chromatin fraction and whole extract, respectively. p97i, p97 inhibitor.

We then examined the chromatin association of replication-related proteins (Fig. 2). In S-phase conditions (−M-extract, +M-extract, and +p27), replisome components such as Cdc45, Psf2, claspin, Polε, and Polδ were maintained on chromatin in the absence of ex-dCTP up to 60 min, while their amount reduced over time in the presence of ex-dCTP because of replication progression and termination (Fig. 2*A*, lane 1–4, lane 9–12). When mitotic entry was induced, those replisome components largely dissociated from chromatin after 60 min regardless of fork release with ex-dCTP (Fig. 2*A*, lane 5–8). Mitotic entry was confirmed by chromatin binding of XCAP-E, a subunit of condensin I/II complex, and mobility shifts of Cut5/TopBP1 (Cut5), APC3, and MCM4. It is unclear whether DNA replication had been fully completed in the presence of the ex-dCTP and M-extract. Considering that replisome components dissociated faster in the M phase than in the S phase (Fig. 2*A*, lane 7 & 8 compared with lane 3 & 4, lane 11 &12), it is possible that replisome disassembly might occur at some forks before completion of DNA replication. These results suggest that replisomes are disassembled both at perturbed and unperturbed replication forks during mitotic progression. Here, unperturbed forks can include ongoing forks and replication-completed converged forks.

Next, we examined the requirement of p97 activity for the mitotic replisome disassembly with or without replication stress. When fork stalling was sustained with Ara-CTP, replisome core factors such as Cdc45, Psf2, and Polε persisted on the chromatin at later time points and polyubiquitylation of MCM7 occurred in the presence of p97 inhibitor (p97i) NMS-873, whereas claspin and Polδ dissociation was not affected significantly (Fig. 2*B*, lane 1–8). This was consistent with our previous results (23). The p97i showed almost the same effect for replisome factors at unperturbed forks (Fig. 2*B*, lane 9–16), suggesting that p97 activity plays a common role for replisome disassembly at replication forks during mitosis whether or not replication stress is persistent.

### Ongoing replication forks delay the NEB during mitotic progression

**Figure 3:**
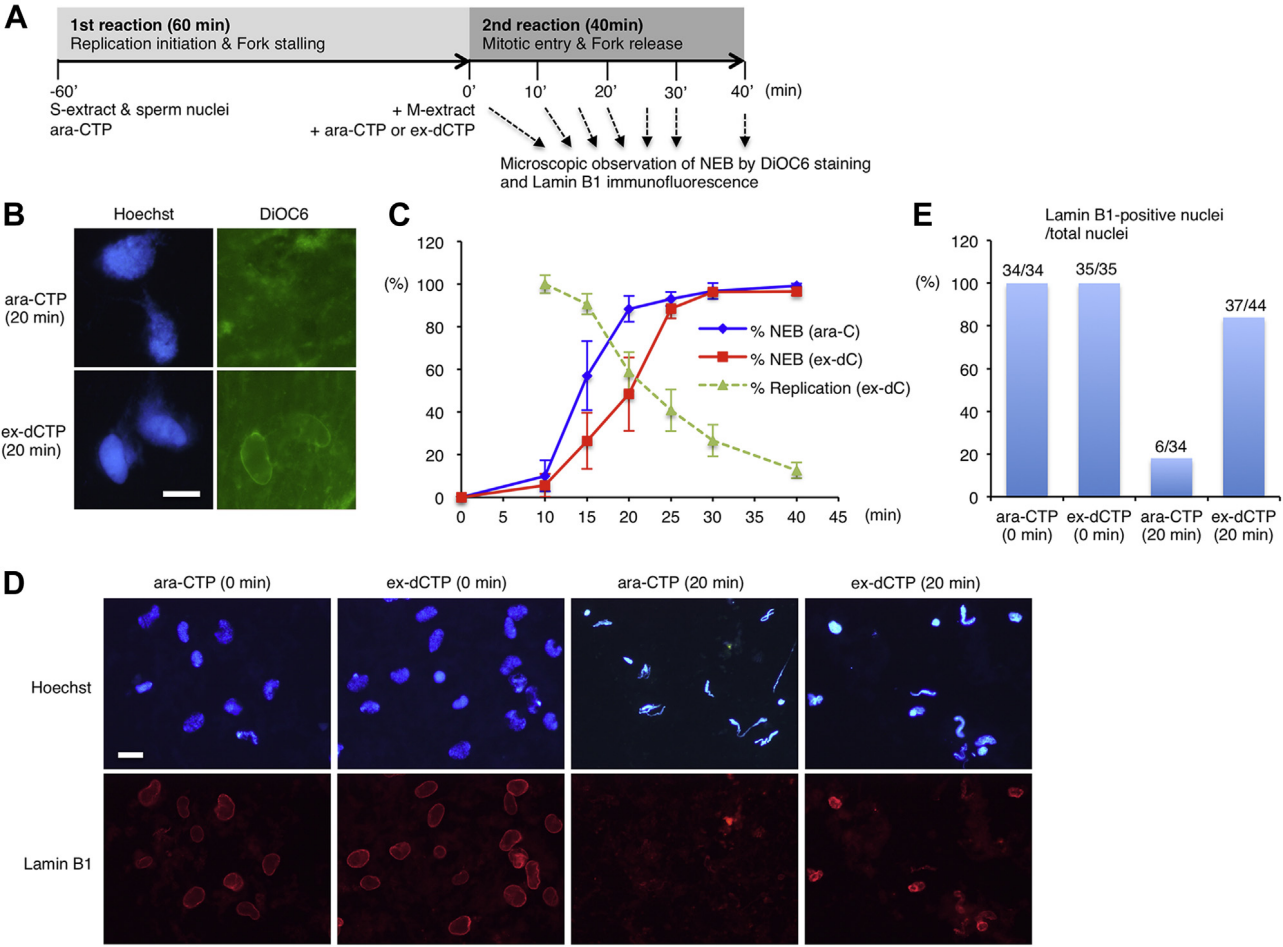
**Ongoing replication forks delay the nuclear envelope breakdown during mitotic progression**. *A*, experimental strategy. *B*, nuclei were fixed after 20 min incubation in the second reaction and stained with Hoechst 33258 for genomic DNA and DiOC6 for nuclear envelope and observed by fluorescence microscopy. Bar, 20 μm. *C*, the same experiment as shown in panel *B* was performed, and the percentages of the nuclear envelope breakdown (NEB) of 50 nuclei were counted at each time point. The average values of three to five independent experiments were plotted on the graph together with each corresponding replication activities obtained in Figure 1, *E*–*F*. Error bar, ± SD. *D*, nuclei were fixed after 0 min and 20 min incubation in the second reaction, subjected to immunofluorescence. DNA was stained with Hoechst 33258. Nuclear lamin B1 was detected with anti-lamin B1 antibody and Alexa 594–labeled secondary antibody. Bar, 20 μm. *E*, each number sets of lamin B1–positive nuclei (numerator) and total nuclei (denominator) were counted at each three different fields, and the percentages of lamin B1–positive nuclei are shown in a graph. DiOC6, 3,3′-dihexyloxacarbocyanine iodide.

Regardless of the relief of replication stress, the addition of an M-extract resulted in mitotic progression in 30 min as evidenced by the band shift of Cut5, APC3, and MCM4 and the chromatin binding of XCAP-E (Fig. 2*A*, lane 5–8). Therefore, we examined if earlier stages of mitotic entry might be affected by fork release. In our experimental setting, the M-extract had a high concentration of already activated mitotic CDK, which acts on interphase nuclei with stalled or ongoing forks. As the NEB is the most obvious structural change during early mitotic entry, we monitored the rate of the NEB progression by microscopic observation of 3,3′-dihexyloxacarbocyanine iodide–stained nuclei (Fig. 3). Intriguingly, we found that the NEB was delayed when fork stalling was released by ex-dCTP as compared with when fork stalling was prolonged by Ara-CTP (Fig. 3, *B*–*C*). After mitotic induction, less than 10% of nuclei underwent the NEB at 10 min and more than 90% at 30 min, in both cases of fork release and stalling. However, a significantly lower percentage of nuclei underwent the NEB between 10 min and 25 min in the presence of ex-dCTP, with 27 to 57% at 15 min, and 48 to 88% at 20 min. Consistently, immunofluorescence also showed a larger proportion of lamin B1–positive nuclei in the presence of ex-dCTP (Fig. 3, *D*–*E*). These results indicate that ongoing replication forks, rather than stalled forks, delay the timing of the NEB during mitotic progression that was induced by the M-extract.

**Figure 4:**
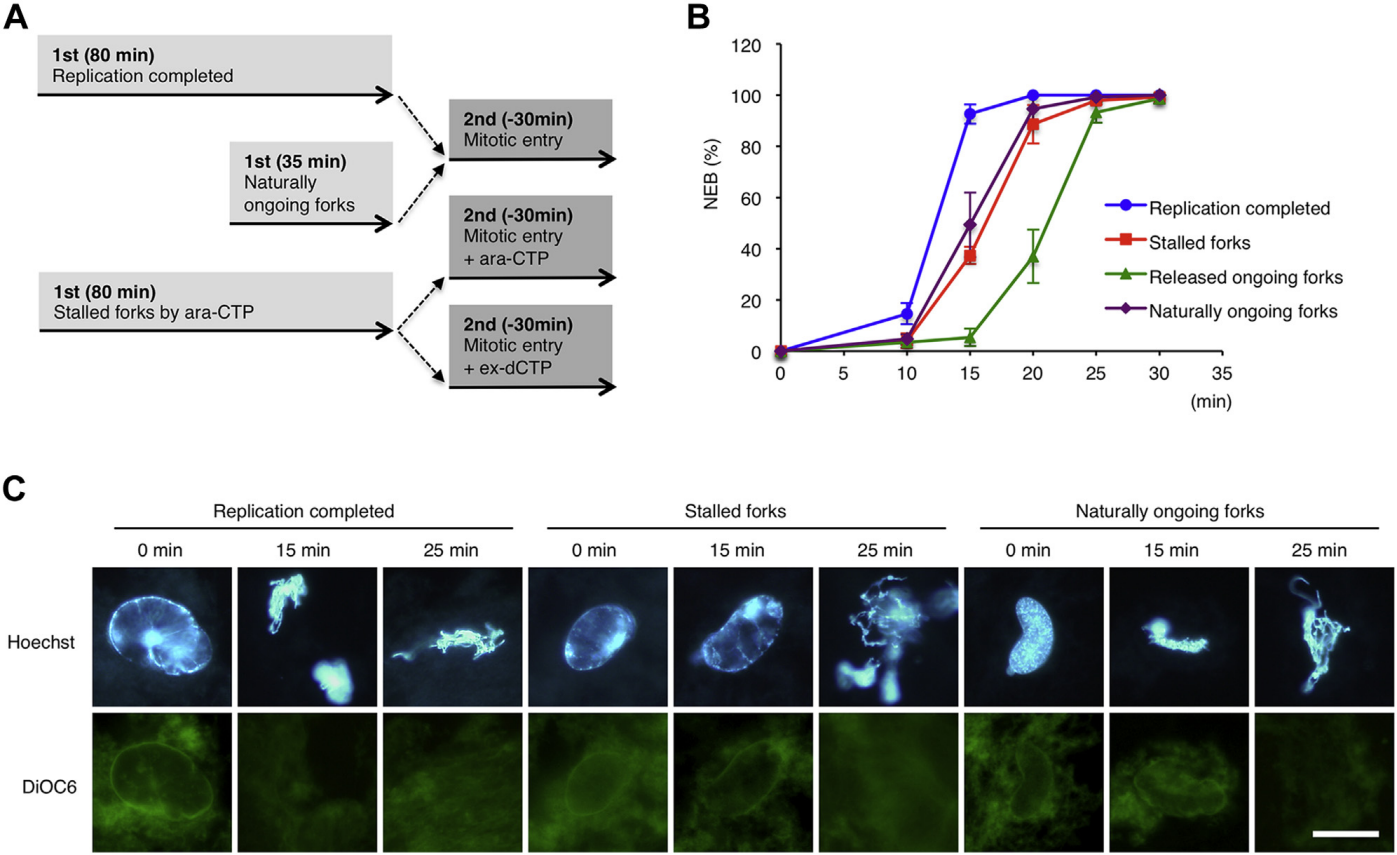
**The kinetic and morphological comparison of the nuclear envelope breakdown between replication-completed nuclei, nuclei with stalled forks, and nuclei with released and naturally ongoing forks**. *A*, experimental strategy. Without replication stress, sperm nuclei generally replicate their genomic DNA in 30 to 60 min in the S-phase egg extract. Therefore, there are naturally ongoing forks at 35 min and no forks at 80 min. Stalled forks are stabilized at 80 min in the presence of Ara-CTP. Then, mitotic entry was induced in the presence or absence of Ara-CTP or ex-dCTP, and nuclei were fixed at each time point and observed by microscopy. *B*, the time courses of the NEB rates were determined as in Figure 3. The same experiments were repeated three times. Error bar, ± SD. *C*, the pictures of nuclei representative at each time point. The genomic DNA and nuclear envelope were stained with Hoechst 33258 and DiOC6, respectively. Bar, 20 μm. Ara-CTP, Ara-cytidine-5′-triphosphate; DiOC6, 3,3′-dihexyloxacarbocyanine iodide.

It was shown that stalled forks also delay the NEB on mitotic entry in *Xenopus* cycling egg extract (34, 35) or interphase extract supplemented with recombinant cyclin B (36). Therefore, we compared the timing of the NEB induced by the M-extract among nuclei with no replication forks (replication completed), with stalled forks by Ara-CTP, with released ongoing forks by ex-dCTP, and with naturally ongoing forks (Fig. 4). Here, “naturally ongoing forks” means that they have not been exposed to exogenous replication stress and are different from “released ongoing forks.” Consistent with the previous studies, the NEB was delayed in the presence of stalled forks to a greater degree, when compared with no replication forks. Naturally ongoing forks did not show further delay in the NEB, but their effect was comparable with stalled forks (Fig. 4*B*). These results indicate that not only stalled forks but also ongoing forks have an inhibitory effect on mitotic progression. Released ongoing forks caused a greater NEB delay than both stalled forks and naturally ongoing forks. This difference between released and naturally ongoing forks might be explained by the difference of total fork numbers or the residual checkpoint activity that works additively for a short while after fork release. In addition, we often observed shrunken chromosomes within the nucleus before the NEB in the presence of ongoing forks (Fig. 4*C*, naturally ongoing forks, 15 min) but not before the NEB in the presence of stalled forks, implying that different mechanisms are responsible for the NEB delay.

### Reducing the number of replication forks dose not affect the timing of the NEB

**Figure 5:**
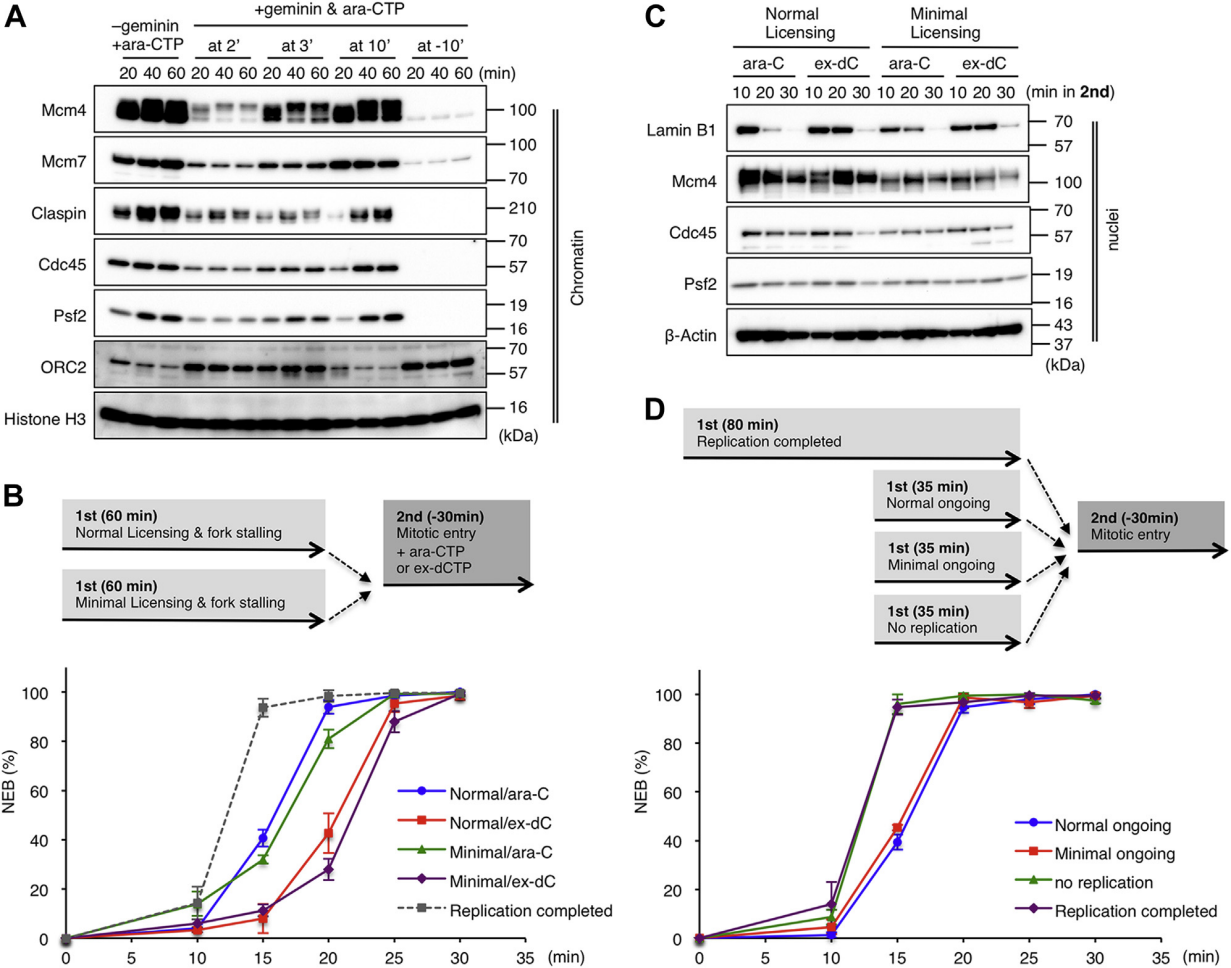
**Reduced number of forks is sufficient for delaying the nuclear envelope breakdown**. *A*, minimal licensing system. After sperm nuclei were mixed with the S-phase extract containing Ara-CTP, recombinant geminin was added at 2, 3, and 10 min (at 2′, at 3′, and at 10′) and further incubated for a total of 20, 40, and 60 min, and the chromatin fractions were isolated and subjected to immunoblotting. As a control, geminin was preincubated for 10 min before adding sperm nuclei (at −10′). *B*, *top*, experimental strategy. Normal or minimal licensing nuclei (+geminin at 2 min) with stalled forks were driven to enter into mitosis with or without fork release. *B*–*D*, *bottom*, the time courses of the average NEB rates in three independent experiments are plotted in the graph. Error bar, ± SD. *C*, nuclear fractions were isolated at each time point and subjected to immunoblotting. β-Actin served as a loading control. *D*, *top*, experimental strategy. Mitotic entry was induced in replication-completed nuclei (80 min), normal licensing nuclei with naturally ongoing forks (normal ongoing, 35 min), minimal licensing nuclei with naturally ongoing forks (minimal ongoing, 35 min), and nuclei with no replication (+geminin at −10 min). Ara-CTP, Ara-cytidine-5′-triphosphate.

We then examined whether the number of replication forks may affect the NEB delay by using the minimal licensing system (37). When sperm nuclei are incubated in the S-extract, the number of pre-RCs gradually increases during the first 15 min, the period before the NE formation. Geminin is a factor that associates with Cdt1 and inhibits pre-RC formation, or “replication licensing.” It is known that the number of pre-RCs can be manipulated by adding exogenous geminin at different times (37). When recombinant geminin was preincubated with the S-extract, pre-RC formation was almost completely suppressed as indicated by the defective chromatin loading of Mcm4 and Mcm7 (Fig. 5*A*, at −10′). In contrast, the addition of geminin at 10 min had a small effect on pre-RC formation (Fig. 5*A*, at 10′). When added at 2 or 3 min, the loading of Mcm4 and Mcm7 was partially suppressed, causing a reduced number of pre-RCs and subsequently reduced number of stalled forks as indicated by the reduced chromatin association of claspin, Cdc45, and Psf2 (Fig. 5*A*, at 2′, at 3′). This situation is called “minimal licensing” because these reduced number of pre-RCs can support normal kinetics of DNA replication progression in the absence of replication stress (37). It was shown that the addition of geminin at 2 min expands interorigin distances about 3-fold (38), indicating the number of replication forks is reduced to approximately one-third.

We compared the timing of the NEB among replication-completed nuclei, normal licensing nuclei, and minimal licensing nuclei by adding geminin at 2 min (Fig. 5*B*). In both normal and minimal licensing, fork stalling caused a similar delay in the NEB, which was further extended by fork release to a similar extent. This result was supported by immunoblotting, which showed that nuclear Lamin B1 was maintained at later time points in the presence of ex-dCTP in both cases (Fig. 5*C*). We also compared the NEB timing among replication-completed nuclei, no-replication nuclei, and normal and minimal licensing nuclei with naturally ongoing forks (“normal ongoing” and “minimal ongoing”) (Fig. 5*D*). When replication initiation was completely inhibited by preincubation with geminin (no replication), the timing of the NEB was almost identical with replication-completed nuclei. Naturally ongoing forks caused a similar NEB delay in both normal and minimal licensing nuclei. Thus, reducing the number of replication forks to about one-third did not affect the timing of the NEB on mitotic entry. These results suggest that reduced number of replication forks is sufficient for delaying the NEB whether or not they are stalled or ongoing.

### Stalled replication forks fail to restart after the NEB

**Figure 6:**
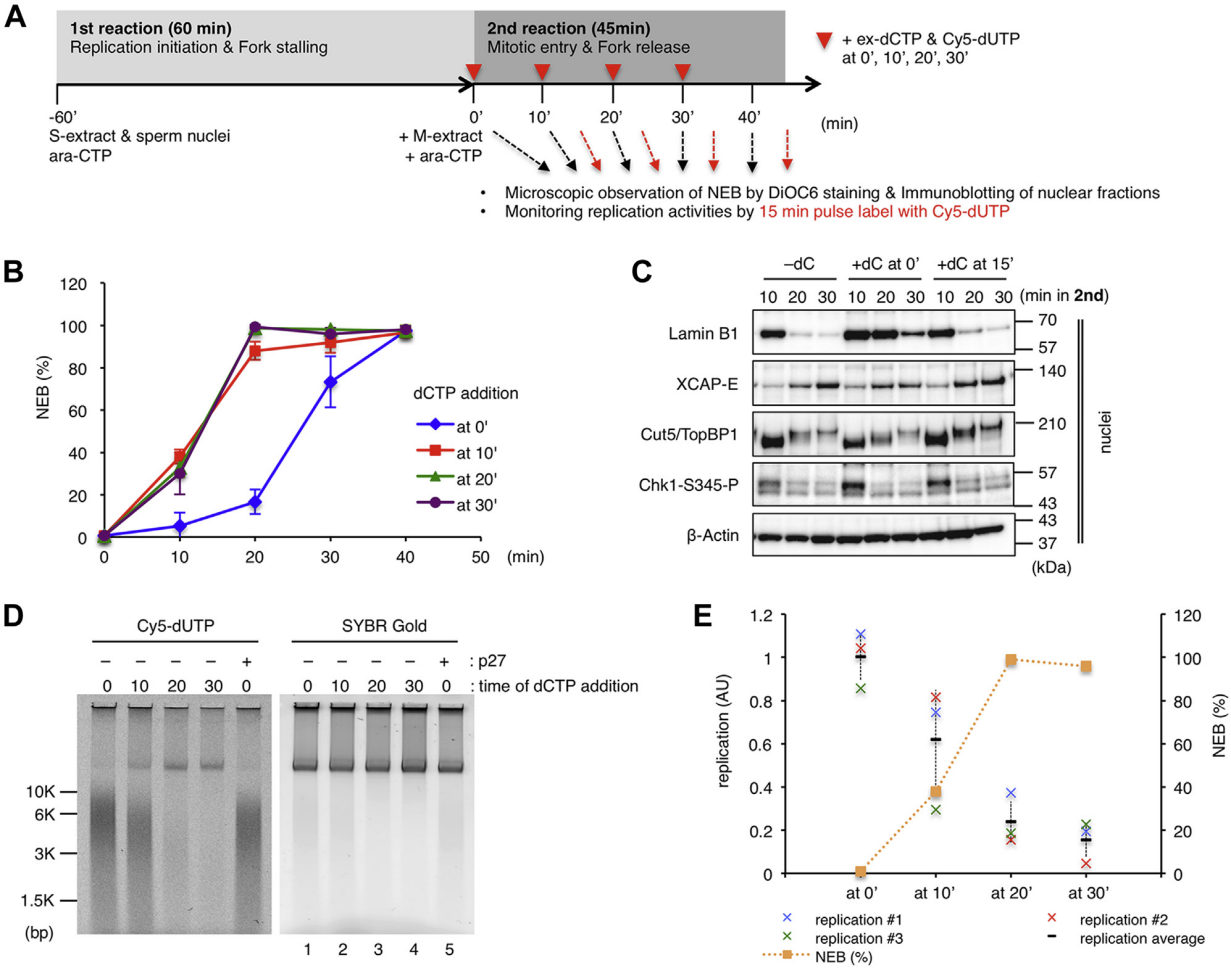
**Stalled replication forks fail to restart after the nuclear envelope breakdown**. *A*, experimental strategy. *B*–*D*, after the first reaction, an M-phase extract and Ara-CTP were added to the reaction mixture and then equally divided into four (*B* and *D*) or three pieces (*C*), to each of which excess amount of dCTP (ex-dCTP, dC) was added at 0, 10, 20, and 30 min (*B* and *D*) (at 0′, at 10′, at 20′, at 30′) or at 0 and 15 min (*C*) (at 0′ and at 15′) in the second reaction. *B*, the time courses of the average NEB rates of each sample were measured as in Figure 3 and shown in the graph. The same experiments were repeated three times. Error bar, ± SD. *C*, nuclear fractions were isolated at each time point and subjected to immunoblotting. The samples without excess dCTP addition were also prepared (−dC). *D*, in the second reaction, Cy5-dUTP was added at the same time with ex-dCTP and further incubated for 15 min, and the genomic DNA was isolated and subjected to 0.8% TAE agarose gel electrophoresis. The same experiments were repeated three times, and the relative replication activities are plotted in the graph in panel *E* together with the NEB rate at the time when ex-dCTP was added. Error bar, ± SD. Ara-CTP, Ara-cytidine-5′-triphosphate.

There seems to be an inverse correlation between the NEB rate and the replication activity in case of fork release (Fig. 3*C*). To clarify if stalled forks can restart after the NEB, we shifted the timing of ex-dCTP addition, or fork release, and monitored NEB rates and replication activities (Fig. 6). When ex-dCTP was added at more than 10 min after mitotic induction, there was no clear difference between the timings of the NEB (Fig. 6*B*, at 10′, at 20′, at 30′). As with the results in Figure 3, the addition of ex-dCTP at the beginning (Fig. 6*B*, at 0′) delayed the timing of the NEB. Correspondingly, the immunoblotting results also show the presence of nuclear lamin B1 at later time points than when ex-dCTP was not added or added at 15 min (Fig. 6*C*). We measured the replication activities for 15 min after fork release (Fig. 6, *D*–*E*). When released at 0 min, immature replication products were broadly distributed between 3 and 10 Kbp, and similar products were obtained in the S-phase control reaction with p27 (Fig. 6*D*, lanes 1 and 5). When released at 20 or 30 min, at which point more than 90% of nuclei had already undergone the NEB, only 15 to 25% of the replication activities were obtained, and a major replication product is beyond 10 Kbp (Fig. 6, *D*–*E*). The release at 10 min, when the NEB rate was about 40%, gave an intermediate result between those of releases at 0 min and at 20 or 30 min. We further investigated the time course of nascent DNA maturation after fork release with alkaline agarose gel (Fig. S1). In the S-phase control with p27, the size of nascent DNA fragments gradually became larger from about 1 to 2 Kb at 5 min to 4 to 10 Kb at 20 min. Similar results were obtained in case of release at 0 min on mitotic entry. However, this pattern of nascent DNA maturation was not observed in the case of release at 25 min after mitotic entry, and Cy5-dUTP seemed directly incorporated into large-size genomic DNA greater than 10 Kb, indicating that DNA repair synthesis occurred. These results suggest that stalled forks can restart DNA replication by replisome before the NEB. After the NE and replisome are disassembled, stalled forks can no longer restart and would collapse and may be repaired by the MiDAS pathway.

The inability of stalled forks to restart may be simply attributed to the absence of replisomes rather than the NEB itself. Therefore, we examined if DNA replication can restart when replisome disassembly was inhibited using a ubiquitin-K0 mutant, in which all the lysine residues are replaced with arginine (Fig. S2). Although replisome components should be maintained on chromatin in the presence of ubiquitin-K0 mutant after mitotic progression (23), we did not detect efficient restart activity with ex-dCTP addition, indicating that those preserved replisomes were not sufficient to support replication restart. These results suggest that an intact replisome and NE are prerequisites for the restart of replication.

### Wee1/Myt1 kinases are required for delaying the NEB in the presence of ongoing forks during mitotic entry

**Figure 7:**
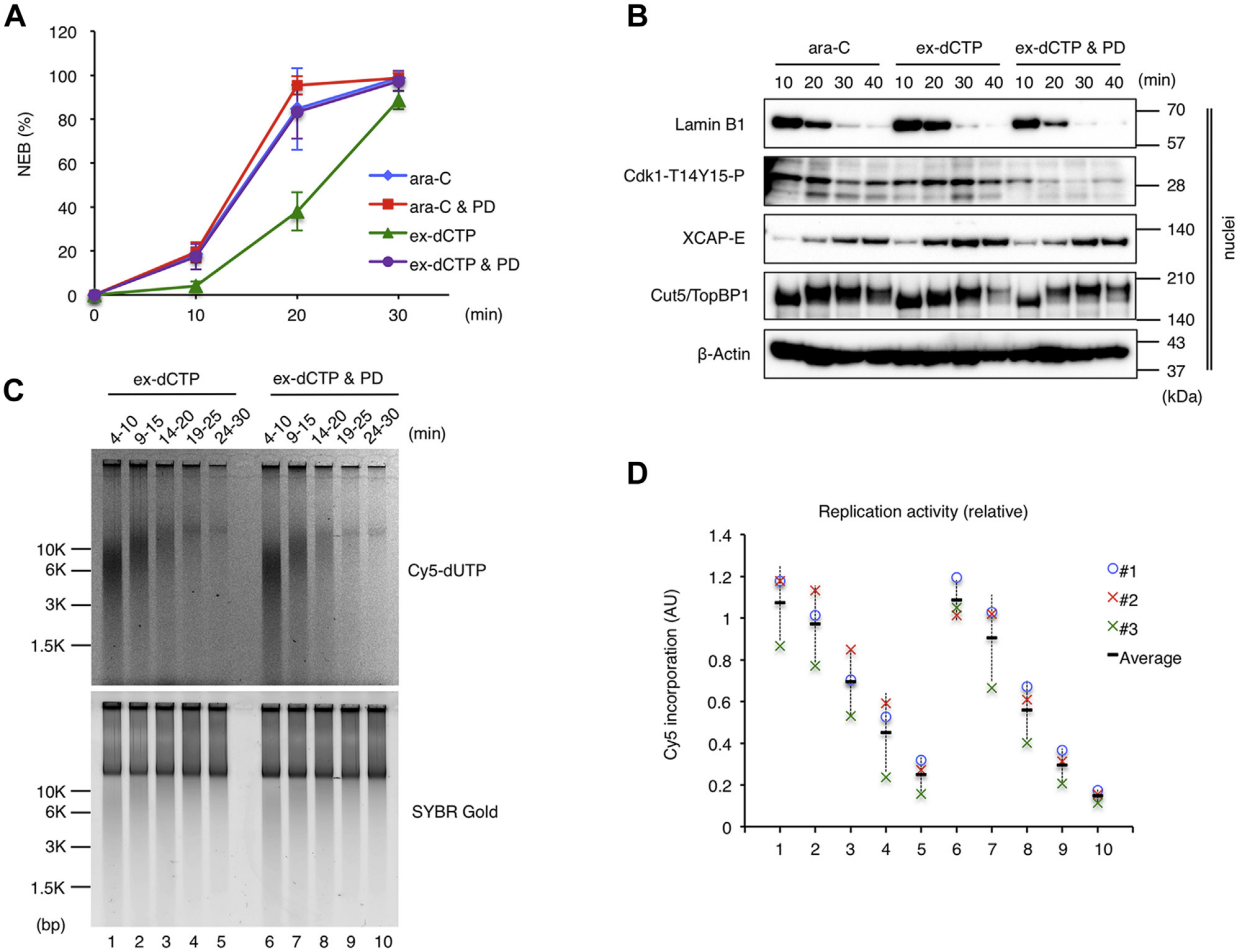
**Wee1/Myt1 kinases are required for delaying the nuclear envelope breakdown (NEB) in the presence of ongoing forks during mitotic entry**. The same experiment as in Figure 3 was performed in the absence or presence of Wee1/Myt1 kinase inhibitor PD166285 (PD). *A*, the average NEB rates in three independent experiments are shown in the graph. Error bar, ± SD. *B*, immunoblotting of nuclear fractions. *C*–*D*, the replication activities at each time point were monitored by 6 min pulse labeling with Cy5-dUTP, and the relative values in three independent experiments are plotted in the graph.

We wondered which signaling pathway is involved in the delay of the NEB in response to replication restart during mitotic entry. In our experimental setting, the ATR-Chk1 pathway was activated in response to fork stalling in the first reaction, and this pathway may have had some contribution to the delay of the NEB in the second reaction before inactivation. However, if only the ATR-Chk1 pathway is involved in the delay of the NEB, stalled forks should have a more significant effect in delaying the NEB than released ongoing forks. It is known that Wee1 and Myt1 kinases keep mitotic CDK inactive by inhibitory phosphorylation to Thr14 (T14) and Thy15 (Y15) until the G2- to M-phase transition (13, 14). Thus, we examined the requirement of Wee1/Myt1 kinase activities for the NEB delay using a chemical inhibitor PD166285 (PD) (Fig. 7) (39). The addition of PD in the presence of ex-dCTP restored the timing of the NEB, whereas PD did not further accelerate the timing in the presence of Ara-CTP (Fig. 7*A*). Accordingly, PD treatment suppressed the phosphorylation of Cdk1 at Thr14 and Tyr15 in nuclei and decreased the amount of nuclear Lamin B1 in the presence of ex-dCTP as fast as in the presence of Ara-CTP (Fig. 7*B*). Pulse labeling with Cy5-dUTP shows that there was no clear difference between the replication activities and products in the presence and absence of PD treatment, excluding the possibility that restart of stalled forks is affected by PD treatment (Fig. 7, *C*–*D*). These results indicate that Wee1 and/or Myt1 kinase activities are required for delaying the NEB during mitotic entry in the presence of ongoing replication forks.

## Discussion

**Figure 8:**
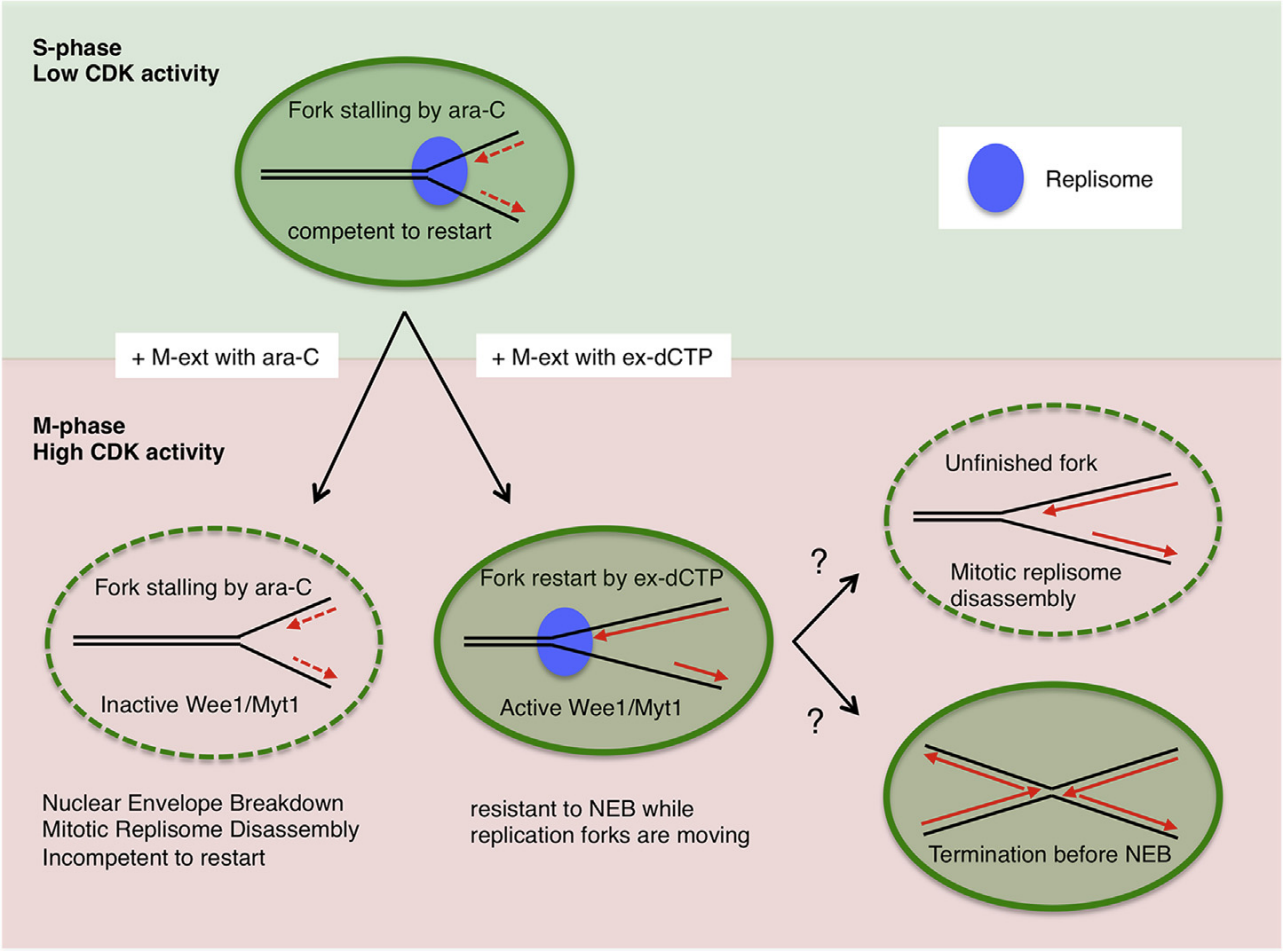
**Replication forks arrested by Ara-CTP are kept competent to restart in the S phase**. Without excess dCTP, mitotic entry drives the nuclear envelope breakdown (NEB) and replisome disassembly, rendering stalled forks unable to restart. In contrast, DNA replication efficiently resumes with excess dCTP while the NEB is delayed in a Wee1/Myt1–dependent manner. It is unclear whether released forks accomplish DNA replication or are subjected to mitotic replisome disassembly on the way. Ara-CTP, Ara-cytidine-5′-triphosphate.

It has long been assumed that DNA replication and mitosis are incompatible and both events never happen simultaneously in eukaryotic cells. Nevertheless, there is increasing evidence that under-replicated genomic DNA is subjected to MiDAS, which offers some sort of final defense against genome instability caused by replication stress before cell division (28, 40). However, it has not been addressed how stalled replication forks are processed during cell-cycle progression from the S phase to the M phase before MiDAS operates. In this study, we revealed that stalled replication forks retain the ability to restart during early mitosis but lose this ability after the NEB during mitotic progression and that ongoing replication forks, if they exist during early mitosis, delay the NEB in a Wee1/Myt1–dependent manner (Fig. 8). This mechanism of delaying the NEB seems distinct from the stress-responsive conventional checkpoint because the former works when replication stress is relieved; it may be linked to an intrinsic brake that ensures the coupling of mitosis to the completion of DNA replication (41, 42).

Although it is unclear exactly at which point during mitotic progression stalled forks become incompetent to restart, it is known that the NE is essential for maintaining the ability to restart. In our experiment, DNA replication could no longer restart efficiently when most nuclei had undergone the NEB. Considering that the nuclear structure is dispensable for replisome assembly and replication progression in a *Xenopus* nucleoplasmic extract system (43), the NE itself may not be directly required for the replication restart in early mitosis. Rather, it is possible that the NE protects stalled forks from being exposed to mitotic cytosol, which contains high concentrations of active mitotic CDK that would promote mitotic replisome disassembly at the stalled forks (23, 24, 25). We utilized a ubiquitin mutant to preserve replisomes on chromatin after mitotic entry, but it was not sufficient to regain the ability to restart (Fig. S2), suggesting that replisome loss is not the sole reason for the inability to restart. Mitotic CDK may also activate nucleases such as Mus81, resulting in the irreversible inactivation of stalled forks (44, 45). We found that even when replication restart became impossible after the NEB, residual DNA synthesis did occur (Fig. 6 and S1), which might correspond to MiDAS activity. It may be interesting to examine the requirement of known factors such as Mus81, Rad52, and PolD3 for this residual activity.

We also found that replication restart temporally halts the NEB but does not cancel it. Whether replication restart is allowed or not, replisomes are disassembled in the end. It is known that the E3 ligase TRAIP and p97 are required for mitotic replisome disassembly at stalled forks, which in turn promotes MiDAS (30). Although p97 was commonly required for replisome disassembly in both cases of fork stalling and fork release (Fig. 2), it is possible that there are some differences in the mechanisms that deal with stalled forks, ongoing forks, and converged forks during mitosis. Our result also shows that ubiquitylated MCM7 accumulated at first, but disappeared later, together with Cdc45 and GINS, even in the presence of a p97i, suggesting a p97-independent pathway for mitotic replisome disassembly (23). This p97-independent disassembly might be simply due to fork processing by nucleases that were activated by mitotic CDK.

It is well established that nuclei with stalled forks delay the NEB timing in an ATR-Chk1 pathway-dependent manner when compared with replication-completed nuclei (34, 35, 36). By contrast, we compared stalled forks with released but unfinished forks, or ongoing forks, and found that released ongoing forks have a more significant effect in delaying the NEB than stalled forks (Figs. 3 and 4). We further demonstrated that the delay of the NEB upon fork release was dependent on Wee1/Myt1 kinase activity (Fig. 7). Because the NEB delay was not affected by the “minimal licensing” that would be expected to reduce the total fork number to about one-third (Fig. 5), a minimal level of DNA replication may be sufficient for the Wee1/Myt1 pathway to operate. Considering that Wee1 localizes to the nucleus, whereas Myt1 is associated with intracellular membranes (13, 14), it is likely that Wee1 is primarily responsible for the NEB delay in response to ongoing forks.

The kinase activities of Wee1/Myt1 are usually high during the S/G2 phases, and the kinases are inactivated upon mitotic entry by phosphorylation and proteolysis (13, 14). It is unknown whether there is any mechanism for activating the kinase activities of Wee1/Myt1 in the S phase, and it has been thought that their relatively high activities during the S/G2 phases simply reflect protein abundance. Because our results suggest that Wee1/Myt1 activities are high in the presence of ongoing forks for a while even after mitotic induction, it gives rise to two hypotheses that ongoing DNA replication either activates Wee1/Myt1 or inhibits the inactivation pathway of Wee1/Myt1. It was shown that Chk1 positively regulates Wee1 through the association with 14-3-3 proteins, contributing to the NEB delay in the presence of stalled forks in *Xenopus* egg extracts (35). Although it is unclear how far this mechanism contributes to the NEB delay in our situation with ongoing forks, or whether it does at all, ATR-Chk1 would be activated in a different mode from replication stress response. ATR activation is regulated through dual pathways involving separate activators TopBP1 and ETAA1 (12). It was recently demonstrated that the TopBP1 pathway mainly responds to replication stress in the S phase, whereas the ETAA1 pathway prevents mitotic chromosomal abnormalities during unperturbed cell cycle (46, 47). Therefore, it is possible that TopBP1-dependent activation of ATR-Chk1 may have been attenuated upon mitotic entry with stalled forks, whereas the ETAA1-mediated pathway became active upon mitotic entry with ongoing forks, resulting in further extended delay of the NEB because of Chk1-dependent Wee1 activation and Cdc25 inhibition. In future studies, it may be important to clarify the exact mode of action of Wee1/Myt1 after replication fork restart.

It was recently proposed that DNA replication itself functions as an intrinsic brake that determines the timing of mitosis by restricting CDK1 and PLK1 activation *via* Chk1/p38 signaling (42). Our results indicate the importance of Wee1/Myt1 for ongoing DNA replication to function as a brake. Because Wee1 is considered as a potential therapeutic target in various types of cancers (39), further deep insights into the relationship between DNA replication and Wee1 activation would be crucial for developing cancer therapy utilizing Wee1 inhibition.

## Experimental procedures

### *Xenopus laevis* egg extracts and replication restart

S-phase (interphase) and M-phase (CSF-arrested) egg extracts and demembranated sperm nuclei were prepared as described (48, 49). In all of the experiments using egg extracts, the reaction temperature was 23 °C, and the concentration of sperm nuclei was 5,000 nuclei/μl in the first reaction with S-extract and was diluted to 2,500 nuclei/μl in the second reaction when mitotic entry was induced with an equal volume of M-extract. Ara-CTP (Jena BioScience) was used at 200 μM to inhibit replication progression in the first reaction. After failure to release forks in the second reaction, another unit of Ara-CTP was added to maintain the final concentration at 200 μΜ. In the event of releasing forks, twenty-fold amount of dCTP was added. Therefore, the final concentrations of Ara-CTP and excess dCTP were 100 μM and 2 mM in the presence of the M-extract, and 200 μM and 4 mM in the absence of the M-extract, respectively, except that 200 μM of Ara-CTP and 4 mM of excess dCTP in the presence of M-extract in (Fig. 6). Chromatin and nuclear fractions were prepared as described (50, 51) and analyzed by immunoblotting.

### Replication assay

For detection of replication activities, each 10 μl extract sample was incubated with 2 μM of Cy5-dUTP (GE Healthcare) for the appropriate number of times and was diluted with 200 μl of stop buffer (5-mM EDTA, 200-mM NaCl, 0.5% SDS, 20-mM Tris-HCl, pH 8.0) containing 200 μg/ml Proteinase K (Roche) and 10 μg/ml RNaseA (Sigma-Aldrich) and incubated for 2 h at 37 °C. The genomic DNA was purified by phenol/chloroform extraction and ethanol precipitation and electrophoretically separated by 0.8% tris-acetate EDTA (TAE) agarose gel (neutral condition) or 1% alkaline agarose gel (denaturing condition) as described (50) and stained with SYBR Gold (Invitrogen). The alkaline gel was neutralized with PBS before SYBR Gold staining because it is pH sensitive. The signals of incorporated Cy5 (Replication activity) and SYBR Gold (total genomic DNA) were scanned with Typhoon FLA 9000 Gel Imager (GE Healthcare) and quantified with ImageJ software.

### Observation of nuclei with fluorescence microscopy

For detecting the NE, each 2-μl extract sample was gently mixed on a glass slide with 4 μl of the fixing solution (3.7% formaldehyde, 2 μg/ml Hoechst 33258, 50% glycerol, 80-mM KCl, 15-mM NaCl, 15-mM Pipes-KOH, pH 7.2) containing 10 μM of 3,3′-dihexyloxacarbocyanine iodide (Sigma-Aldrich) and squashed under a 22-mm × 22-mm square coverslip.

For detecting replication activity and immunofluorescence, each 10 μl extract sample was diluted with 90-μl of extraction buffer (EB) (100-mM KCl, 2.5-mM MgCl_2_, 50-mM Hepes-KOH, pH 7.5), 11 μl of 37% formaldehyde was then added, incubated at RT for 10 min, 1 ml of EB was added further, which was loaded into a swinging bucket for collecting suspension culture cells (CS-2, Tomy). Nuclei were collected by centrifuge (500*g*, 5 min) onto poly-L-lysine–coated coverslip (IWAKI) through 0.5-ml of EB plus 30% sucrose layer. DNA replication was labeled with 1 μM of CF594-dUTP (Biotium). Nuclear lamin B1 was detected by sequential incubation with anti-lamin B1 antibody (ab16048, Abcam) and Alexa 594 anti-rabbit IgG (Thermo Fisher). The coverslips were washed with TBS-T, PBS, and dH_2_O and mounted on glass slides with 3 μl of SlowFade Gold (Thermo Fisher) containing 2 μg/ml Hoechst 33258.

### Antibodies and reagents

Antibodies to Psf2, XCAP-E, claspin, and Cut5/TopBP1 were prepared as per directions (23, 52, 53). Antisera to Cdc45 and Polε (p60) were provided by H. Takisawa and Y. Kubota (Osaka University). Antiserum to Polδ (p66) was provided by S. Waga (Japan Women’s University). Antibodies to APC3 and Cyclin B2 were provided by S. Mochida (Kumamoto University). The following antibodies were obtained from the indicated companies: MCM7 (sc-9966, Santa Cruz), MCM4 (ab4459, Abcam), lamin B1 (ab16048, Abcam), β-actin (ab8224, Abcam), Phospho-Chk1 (Ser345) (2341, Cell Signaling), Phospho-CDK1 (Thr14, Tyr15) (44-686G, Thermo Fisher). Recombinant His-p27 and His-geminin were prepared as described (50) and used at 100 μg/ml and 50 μg/ml to inhibit CDK activities and replication licensing, respectively. NMS-873 (Sigma-Aldrich) and PD166285 (Sigma-Aldrich) were used at 100 μM and 10 μM to inhibit p97/VCP and Wee1/Myt1 kinase activities, respectively.

## Data availability

All data are contained within the article and accompanying supporting information.

## Conflict of interest

The authors declare that they have no conflicts of interest with the contents of this article.
