# Supplementary material for: Ongoing replication forks delay the nuclear envelope breakdown upon mitotic entry
Source: J Biol Chem. 2020 Nov 23;296:100033. doi: 10.1074/jbc.RA120.015142 (PMC7948514; doi:10.1074/jbc.RA120.015142)
Supplement: Figures S1–S9 [file mmc1.pdf]

Supporting Information for

Ongoing replication forks delay nuclear envelope breakdown upon mitotic entry

Yoshitami Hashimoto* and Hirofumi Tanaka

School of Life Sciences, Tokyo University of Pharmacy and Life Sciences
1432-1 Horinouchi, Hachioji, Tokyo 192-0392, Japan

A

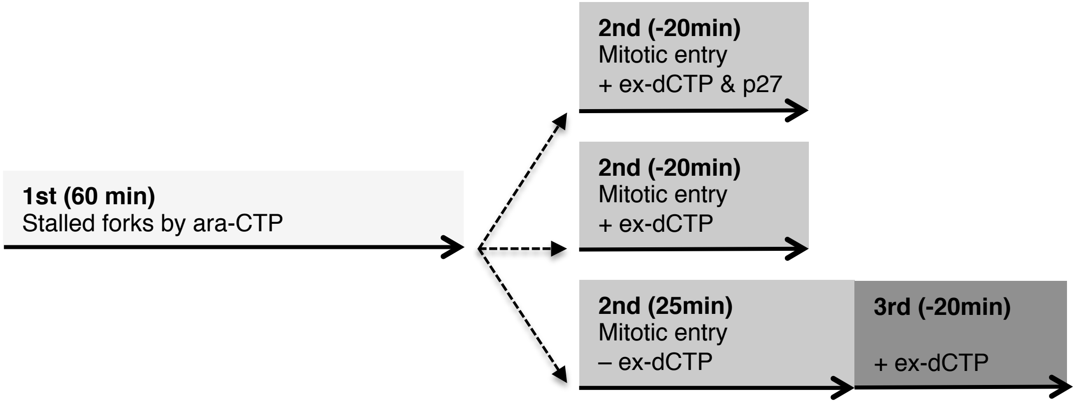

Figure S1. Nascent DNA maturation after fork release upon mitotic entry. (A) Experimental strategy. (B) After the 1st reaction, an M-phase extract and ara-CTP was added to the reaction mixture, then equally divided into three pieces, to each of which Cy5-dUTP and excess amount of dCTP (ex-dC) were added at 0 or 25 min (0', 25') in the 2nd reaction. In the S-phase control, p27 was added at 0 min (+p27). After the release, the genomic DNA was isolated at every 5 min up to 20 min and subjected to 1% alkaline agarose gel electrophoresis, followed by SYBR Gold staining.

B

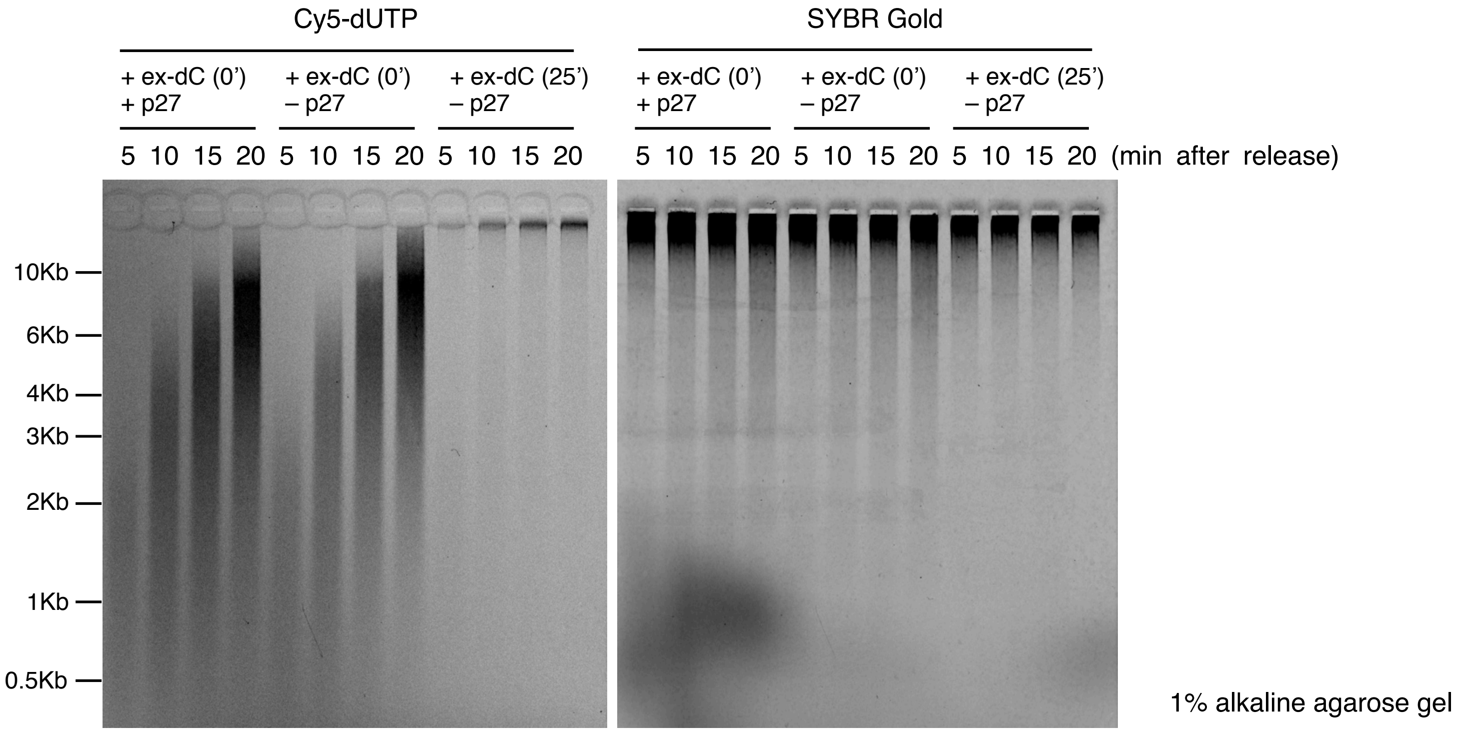

Figure S1

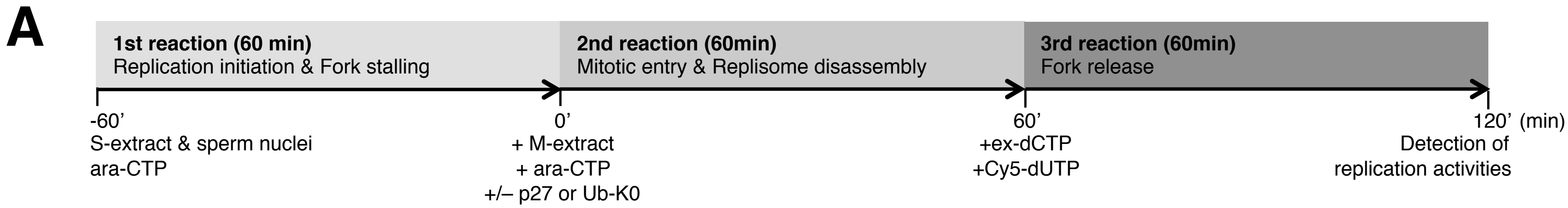

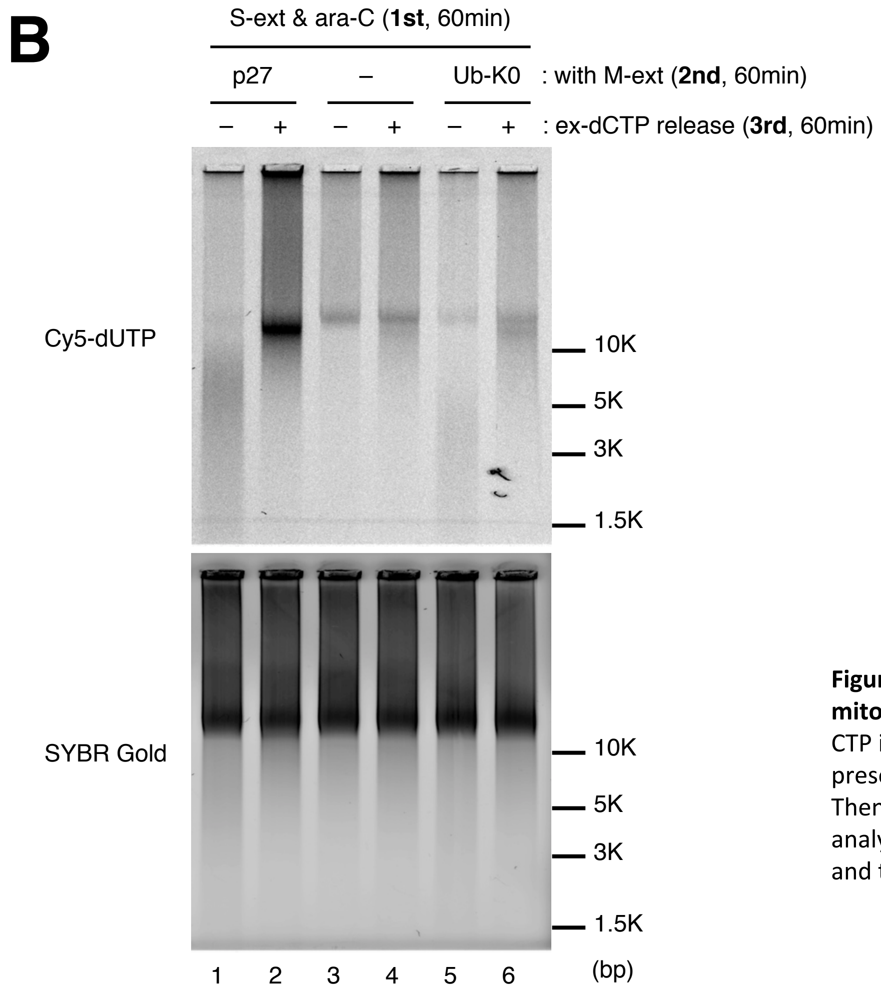

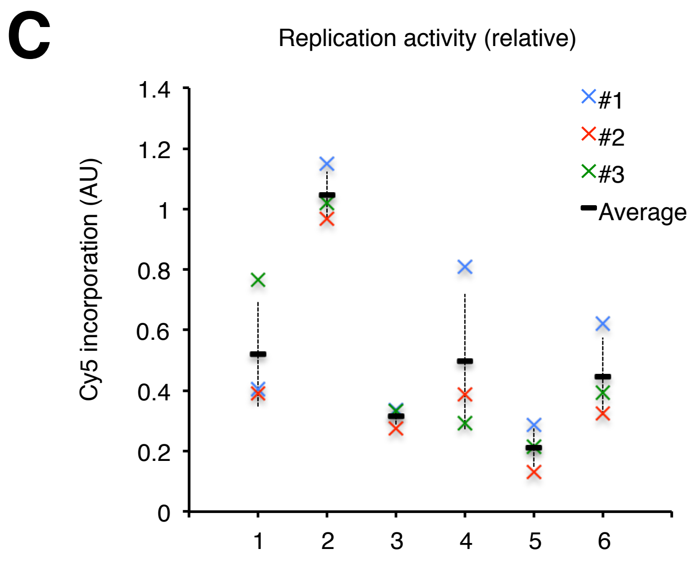

Figure S2. Replisome preservation is not sufficient to support replication restart after mitotic progression. (A) Experimental strategy. After the 1st reaction (fork stalling by ara-CTP in S-phase), mitotic entry was induced with ara-CTP to keep replication stress in the presence or absence of ubiquitin K0 (Ub-KO), which prohibits replisome disassembly. Then, excess dCTP was added to relieve replication stress. (B) Replication activities were analyzed in a similar way as in Fig. 1. The same experiments were repeated three times and the relative values were plotted in the graph (C). Error bar, ± S.D.

Figure S2

Raw images of immunoblots in Figure 2A (Chromatin)

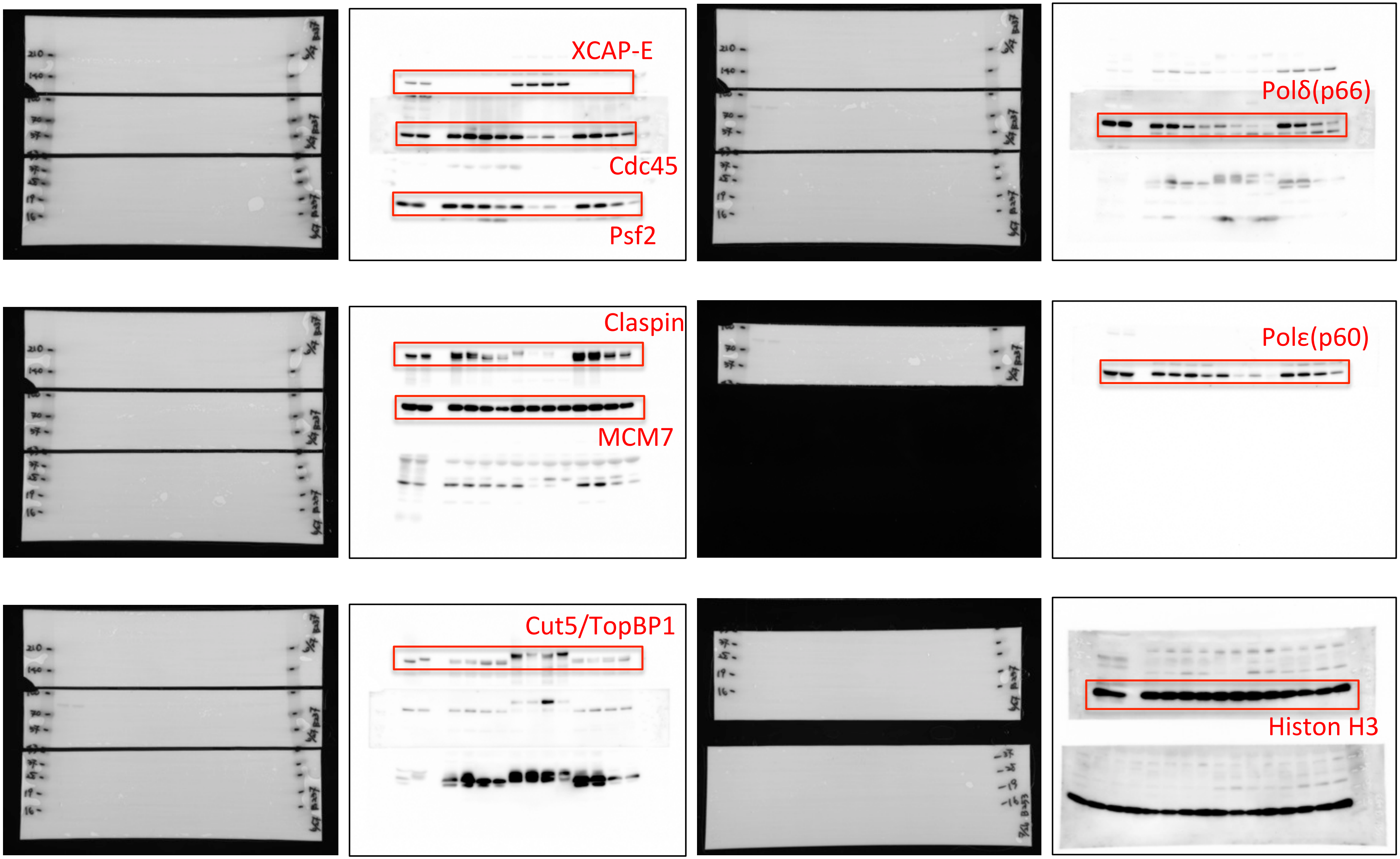

Figure S3

Raw images of immunoblots in Figure 2A (Whole extract)

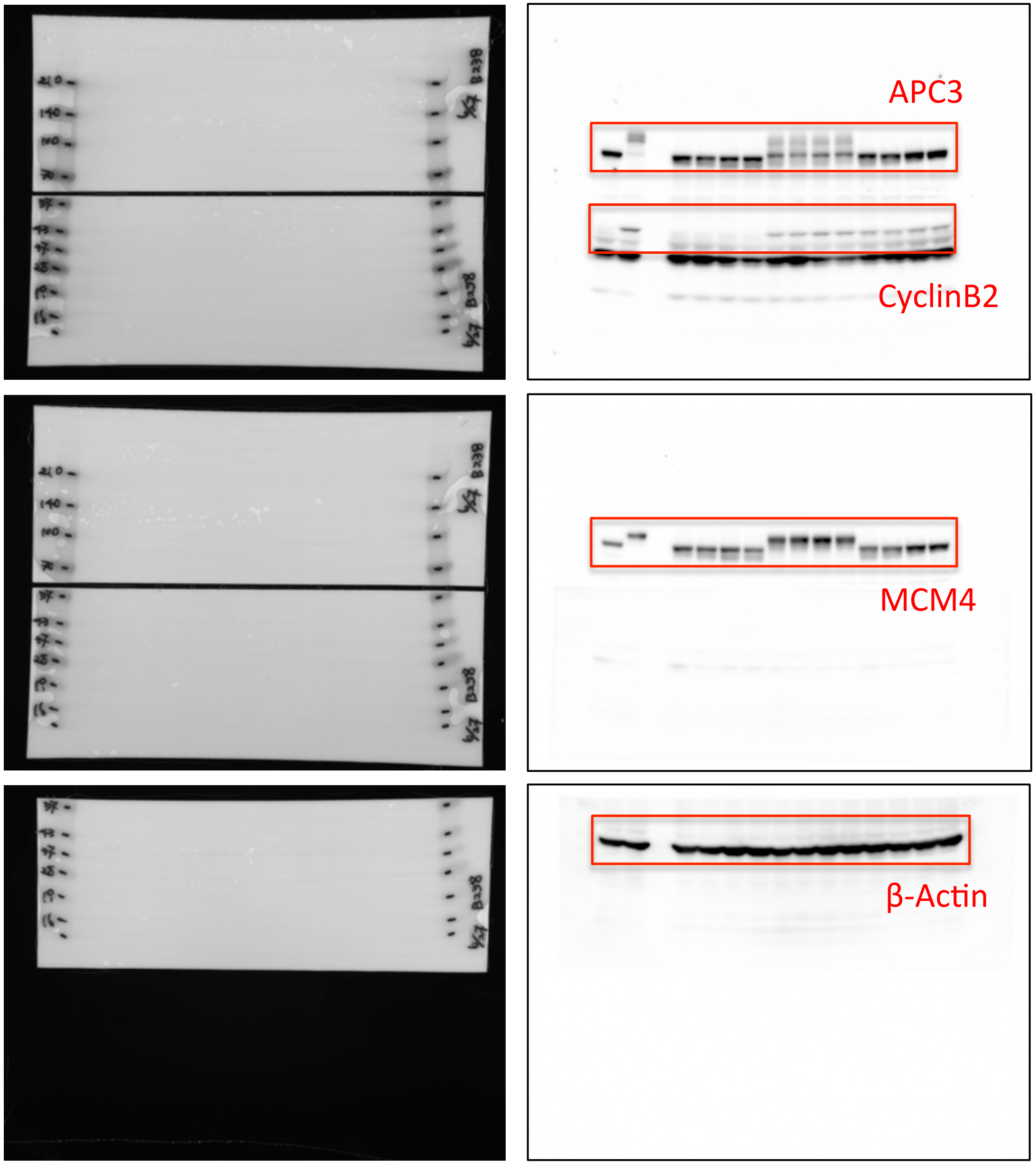

Figure S4

Raw images of immunoblots in Figure 2B (Chromatin)

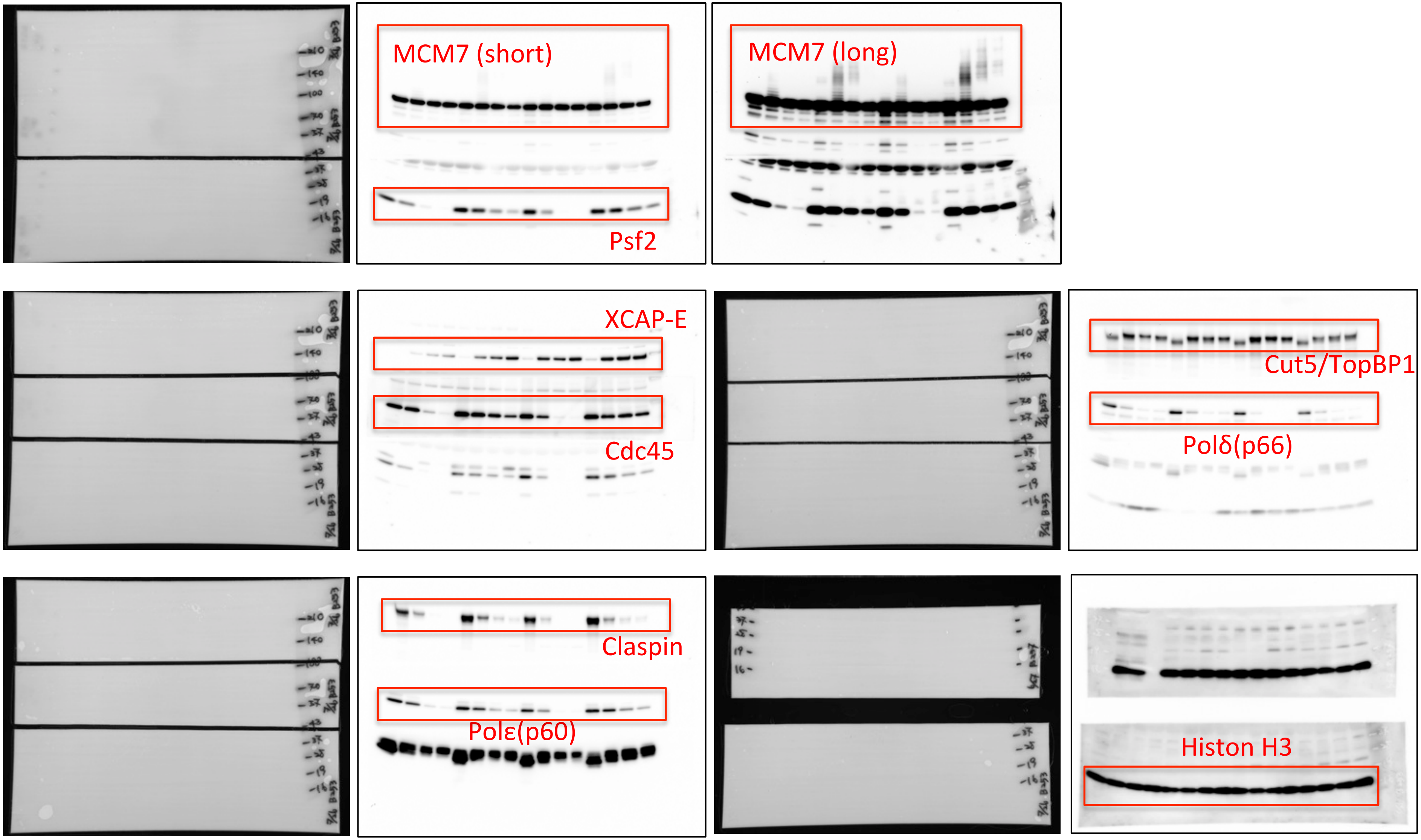

Figure S5

Raw images of immunoblots in Figure 5A (Chromatin)

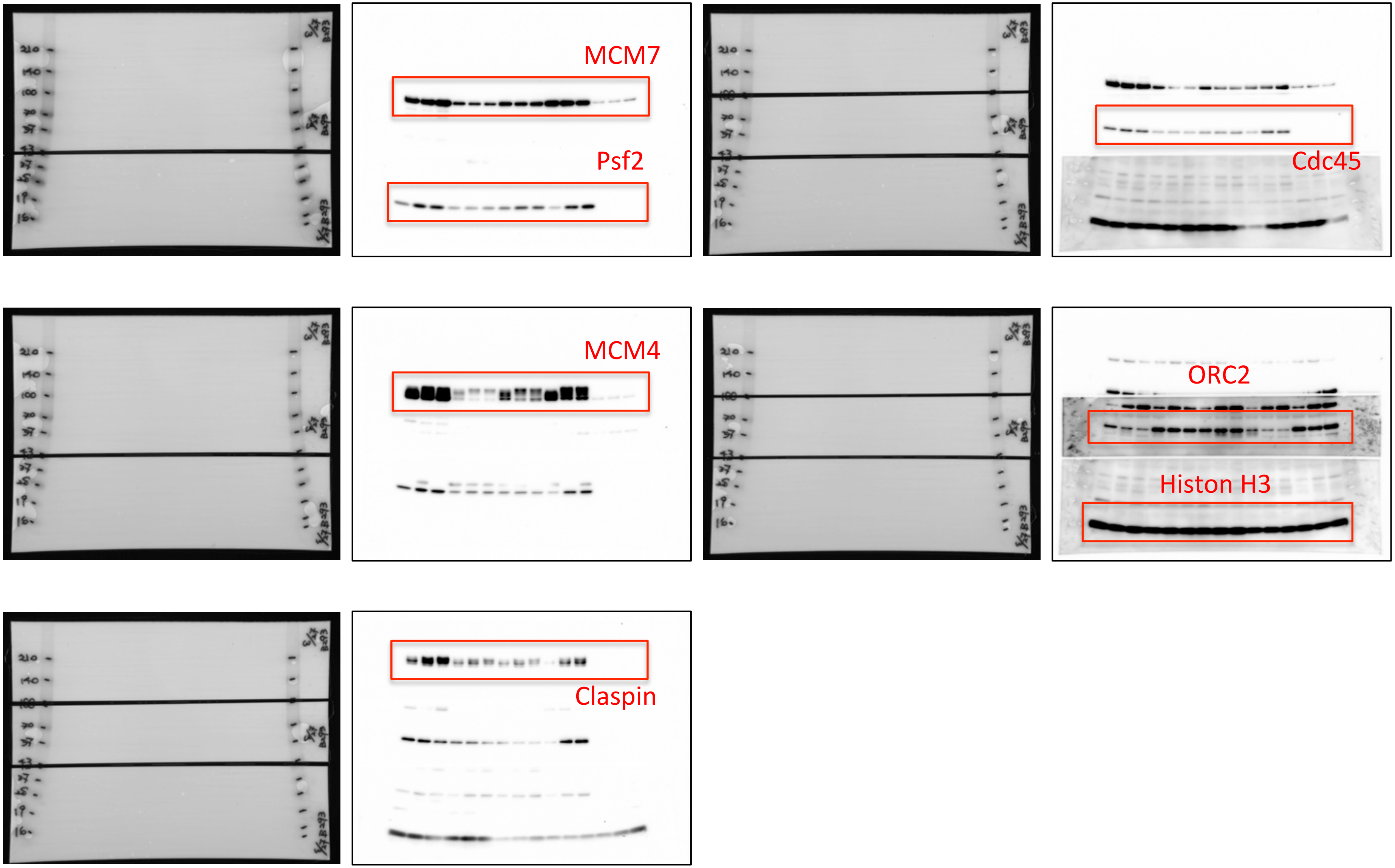

Figure S6

Raw images of immunoblots in Figure 5B (Nuclei)

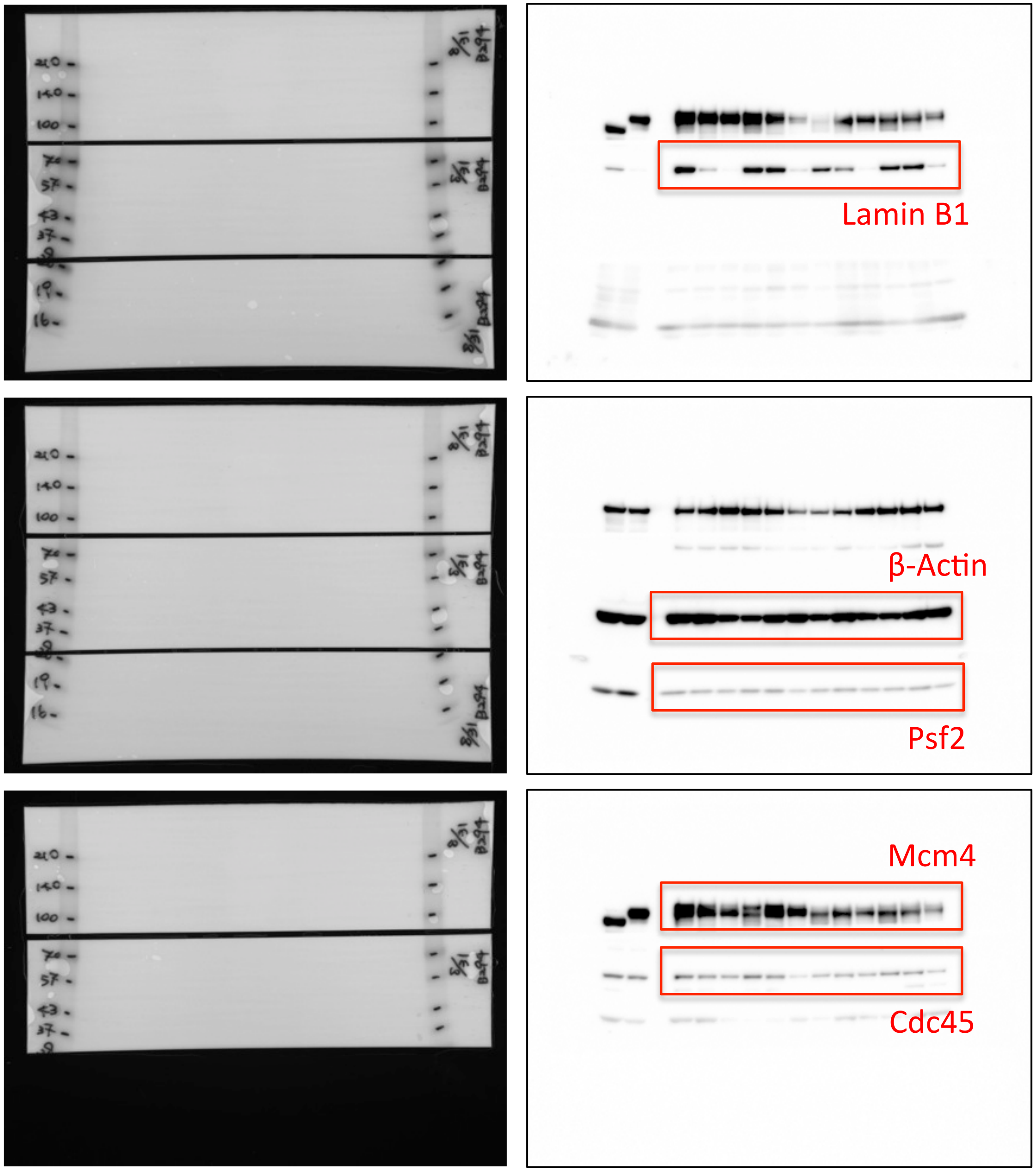

Figure S7

Raw images of immunoblots in Figure 6C (Nuclei)

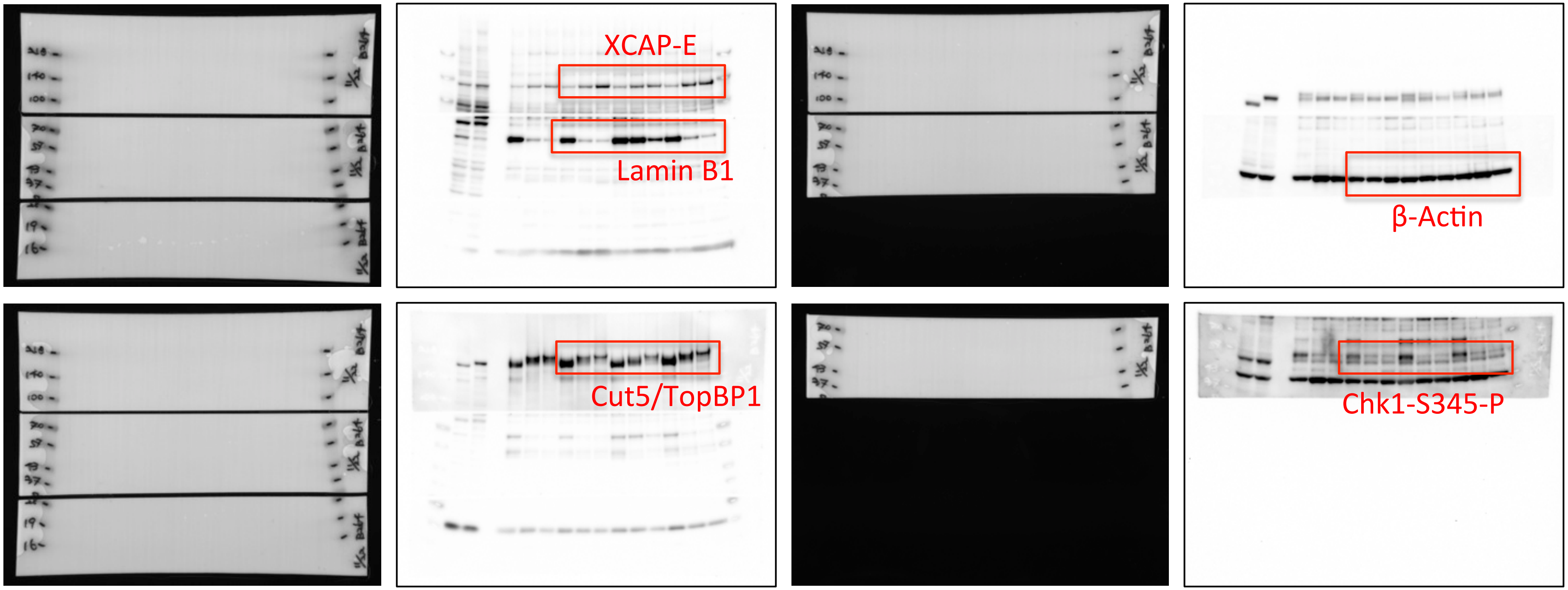

Figure S8

Raw images of immunoblots in Figure 7B (Nuclei)

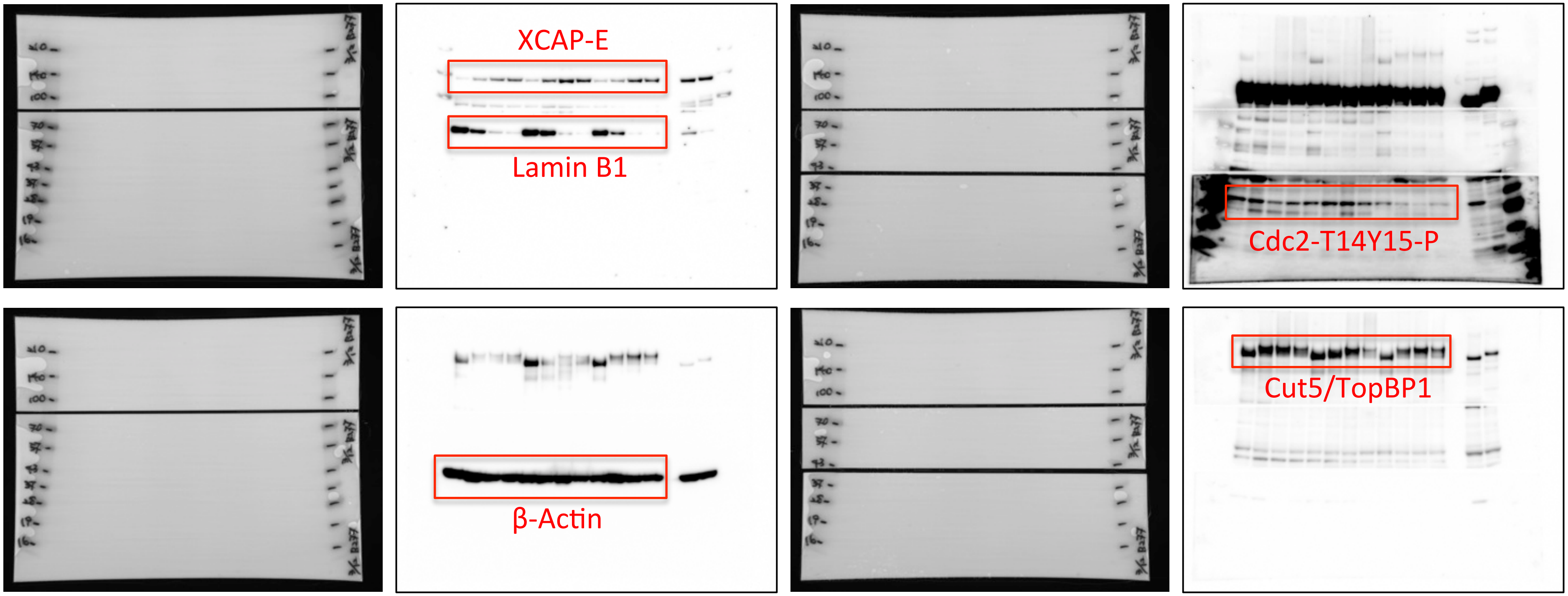

Figure S9
